# The Application of Waste Coal Fly Ash as a Support for a Heterogeneous Catalyst for Biodiesel Production and a Comparison with the Engine Characteristics Using a Homogeneous Catalyst

**DOI:** 10.1021/acsomega.6c04422

**Published:** 2026-10-13

**Authors:** Cherng-Yuan Lin, Yi-Tsai Liu

**Affiliations:** Department of Marine Engineering, 34880 National Taiwan Ocean University, No. 2, Beining Road, Zhongzheng District, Keelung City 202301, Taiwan

## Abstract

Coal fly ash is a waste product of coal-fired boiler combustion. The fly ash consists of fine glass microspheres with a relatively large surface area. This study aims to synthesize catalysts using waste coal fly ash as a support and calcine them with alkaline catalysts. Fly ash with a large surface area was synthesized using sodium methoxide to prepare a heterogeneous catalyst, which was then used to transesterify palm oil to produce biodiesel. Subsequently, engine performance and exhaust emissions from biodiesel combustion were evaluated. The experimental results indicate that biodiesel prepared with the heterogeneous catalyst exhibited lower fuel consumption and brake-specific fuel consumption (BSFC) than biodiesel prepared with the homogeneous catalyst, and it had a higher brake thermal efficiency. The emission concentrations of CO, CO_2_, and NO_X_ are higher in biodiesel produced with homogeneous catalysts, whereas the O_2_ emission concentration from biodiesel prepared with a heterogeneous catalyst is lower than that of homogeneous biodiesel. Moreover, the biodiesel produced using a heterogeneous catalyst consisting of coal fly ash containing sodium methoxide appeared to have a lower excess air ratio and heating value, while exhibiting a higher exhaust gas temperature and fuel/air equivalence ratio. Using waste coal fly ash to synthesize a heterogeneous catalyst improves biodiesel properties, thereby advancing the circular economy.

## Graphical abstract

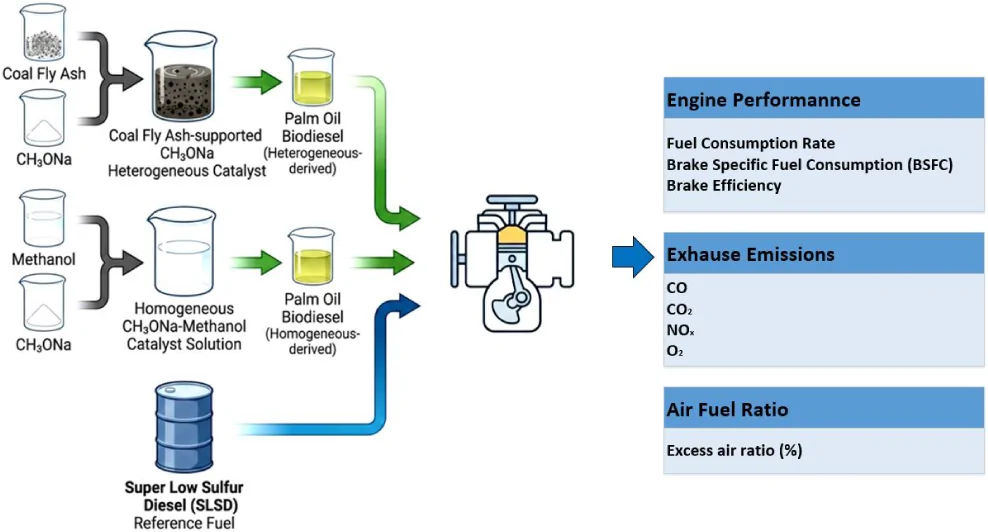

## Introduction

This study aims to evaluate the suitability of coal fly ash, which contains high levels of SiO_2_ and Al_2_O_3_, has a porous structure and high specific surface area, and is a waste byproduct, as a free catalyst support. Furthermore, these characteristics are used to add value to fly ash waste^1^ by adsorbing sodium methoxide, thereby enabling a heterogeneous transesterification reaction among methanol, fly ash, and the raw oil.

Research on the reuse of coal fly ash primarily focuses on its fundamental properties and engineering applications. Notably, studies employing coal fly ash as an adsorbent for pollutant removal from water primarily focus on optimizing control conditions and removal efficiencies for various contaminants, whereas only a limited number of articles examine its applications in the energy sector in depth.

The fly ash employed in this study is produced from coal-fired power plants. The advantages of fly ash include its surface micropores and the fact that the coal fly ash comprises over 80% SiO_2_ and Al_2_O_3_. Hence, it is considered a free catalyst support. The synthesized fly ash/sodium methoxide catalyst is applied to the transesterification reaction of palm oil. The resulting biodiesel product is characterized and compared with biodiesel produced using conventional homogeneous sodium methoxide catalysts. Biodiesel produced using homogeneous and heterogeneous sodium methoxide catalysts was compared in terms of engine performance and exhaust emission concentrations.

Fly ash is the residue left after coal combustion and is an environmental pollutant. Fly ash is predominantly gray in color and exhibits alkaline properties.^2^ Its composition includes phosphorus, potassium, calcium, magnesium, zinc, iron, copper, manganese, boron, and molybdenum.^3^ Its applications are diverse; it can serve as an adsorbent for industrial exhaust gases, remove toxic metals and other inorganic substances from wastewater, and eliminate dyes from wastewater.

The fly ash contains more than 80 wt % of SiO_2_, Al_2_O_3_, and Fe_2_O_3_, with a low lime content, thus classifying it as Class F fly ash.^4^ If the fly ash contains 50–70 wt % of SiO_2_, Al_2_O_3_, and Fe_2_O_3_, with a low lime content, it is classified as Class C fly ash. Class C fly ash can be obtained from lignite and sub-bituminous coal and exhibits binding properties, hardening automatically upon contact with water, whereas Class F fly ash is obtained from anthracite and bituminous coal (commonly known as volcanic ash) and hardens automatically when reacting with calcium hydroxide and water.

The primary difference between Class C and Class F fly ash is that Class F fly ash contains 1–12% calcium, primarily as calcium oxide, calcium sulfate, and vitrified components. In contrast, Class C fly ash contains 30–40% calcium oxide and higher amounts of alkaline substances (such as sodium and potassium) and sulfur oxides. The fly ash used in this study is Class F fly ash.

The annual production of fly ash from coal-fired power plants is substantial, often causing bottlenecks in its disposal. According to the literature, coal fly ash with varying mineral compositions exhibits a unit adsorption capacity for copper ions in water ranging from 0.12 to 1.87 (mg Cu^2+^/g fly ash). The mineral composition of coal ash influences its adsorption capacity for copper ions, with unit adsorption capacities varying by up to 16-fold across coal fly ash samples with different mineral contents.^5^

The primary technology for biodiesel production employs transesterification,^6,7^ which involves mixing oils and fats with short-chain alcohols in the presence of a catalyst to produce fatty acid methyl esters (FAME).^8^ Fatty acid methyl esters are mainly synthesized via ester exchange between triglycerides and methanol.^9^ Transesterification proceeds through three stages: triglyceride → diglyceride → monoglyceride → glycerol.^10^ Factors affecting the conversion rate include the molar ratio of alcohol to oil, catalyst loading, reaction temperature, reaction time, moisture content, and free fatty acid content.^11^

The transesterification process is classified into three categories based on the catalyst type: acidic, alkaline, and enzymatic. Acidic catalysts include sulfuric acid (H_2_SO_4_), sulfonic acid (SO_3_H), and hydrochloric acid (HCl). Alkaline catalysts include sodium hydroxide (NaOH), potassium hydroxide (KOH), sodium methoxide (NaCH_3_O), sodium bicarbonate (NaHCO_3_), and potassium carbonate (K_2_CO_3_), among others. The advantage of the acidic process lies in its ability to esterify free fatty acids simultaneously, thereby offering greater tolerance to feedstock oils. However, it requires significantly longer reaction times and poses a higher risk of corrosion to equipment and containers. Therefore, the most commonly employed industrial process for biodiesel production remains the alkaline method. Its drawback is the stringent limitation on feedstock oil quality, as free fatty acids readily react with the alkaline catalyst to form soap residues, resulting in unnecessary waste. Moreover, excessive soap formation complicates glycerol production and separation. Jariah et al.^12^ observed that when the acid value exceeds 2 mg KOH/g, soap formation with the alkaline catalyst occurs readily, reducing transesterification efficiency and impairing glycerol separation. The primary advantage of the alkaline process is its short reaction time and high methyl ester yield, which can reach 98%. The enzymatic process can simultaneously esterify triglycerides and free fatty acids, effectively addressing the glycerol recovery issue. Disadvantage of the enzymatic process is the high cost of the enzyme itself. Although the conversion rate can reach 99%, the reaction time is too long, and the costs are high, rendering it unsuitable for industrial-scale production. This study employs transesterification with alkaline catalysts to produce biodiesel using both homogeneous and heterogeneous catalysts. Triglyceride is a major compound of the fat or lipid in vegetable oils or animal fats. Triglyceride is mixed with methanol at a molar ratio from 1:3 to 1:9 to carry out transesterification, as shown in Figure 3. At the end of the production process, fatty acid methyl esters accompanied by about 10 wt % glycerol are formed. Transesterification of a triglyceride with methanol is a primary reaction used to produce biodiesel, with glycerol as a byproduct. The process occurs in three consecutive reaction steps.^13^ T, D, M, and G refer to triglyceride, diglyceride, monoglyceride, and glycerol. MeOOR denotes the product of fatty acid methyl esters (i.e., FAME).

The overall reaction stoichiometry of transesterification toward biodiesel production is as follows.^13^

1 T+3 MeOH⇐⇒G+3 MeOOR

Catalysts are generally classified into two types based on their physical state: homogeneous and heterogeneous. Homogeneous-phase catalysts have drawbacks, including difficulties with product separation and recovery, which increase production costs and environmental pollution. Heterogeneous catalysts offer advantages including superior thermal stability, mechanical strength, and ease of product separation. These benefits not only enhance catalyst reusability and product purity but also mitigate environmental pollution. Therefore, heterogeneous catalysts hold greater practical value in the chemical industry.^7^ The primary advantage of solid or heterogeneous catalysts is their ease of separation from the product, enabling catalyst recycling. Current solid catalysts are mainly classified as solid acids and solid bases. Solid acid catalysts are primarily used in biodiesel production. Acid catalysts can catalyze both esterification and transesterification, thereby enabling the use of feedstock oils with high free fatty acid (FFA) content. The advantage of solid-base catalysts lies in their higher reactivity, enabling rapid transesterification under low-temperature, atmospheric-pressure conditions.

The preparation of solid catalysts often employs the impregnation method, which evenly disperses a small amount of crystals, particularly noble metal crystals, onto a porous catalyst support with a large surface area, producing supported noble metal catalysts.^6^ The impregnation method enables the maximal utilization of expensive components. The impregnation method can be classified into two types: the first involves immersing the catalyst support in a large volume of solution, in which the amount of catalyst support and, consequently, the solution concentration vary. The second method involves mixing the catalyst support with an appropriate amount of solution to form a slurry. Multiple impregnation steps are sometimes necessary to achieve the desired metal content.

Heterogeneous catalysts are classified as supported or unsupported. To improve economic efficiency and reduce costs, suitable catalyst supports are commonly employed in the preparation of supported catalysts. The factors to consider for the selection of catalyst support are^8^ (1) porosity, (2) typically high surface area, (3) chemical stability during reaction and regeneration, (4) appropriate mechanical properties including hardness, compressibility, and wear resistance, (5) inertness, and (6) low cost. Solid alkaline catalysts are considered promising for improving the chemical conversion rate of feedstock oil to produce valuable biofuels. The preparation and characteristics of solid alkaline catalysts, such as sodium methoxide supported on waste coal fly ash, remain poorly investigated. Previous applications of waste coal fly ash have mostly been in wastewater treatment; few studies have examined its use in the energy sector, particularly for biodiesel production. The use of coal fly ash in biofuel production can advance the circular economy and significantly reduce industrial waste.

## Experimental Details

### Preparing Palm Oil Biodiesel through Transesterification Using a Heterogeneous Catalyst Carried by Coal Fly Ash

The coal fly ash was collected from a coal-fired boiler in Taoyuan City, Taiwan. The appearance of the coal fly ash is shown in Figure 1. Prior to preparation, coal fly ash was immersed in an aqueous solution of sodium methoxide for 24 h. The fly ash-sodium methoxide composite was then dried and incinerated in a high-temperature furnace to obtain the calcined catalyst. The preparation procedures are described below: (1) The fly ash and sodium methoxide were prepared in proportions of 1.1–6.5 wt % to immerse the fly ash in the sodium methoxide solution for 24 h; (2) The aqueous solution was poured into a porcelain crucible; (3) controlling the temperature of the high-temperature furnace in 4 stages, which were increased to 100 °C at an increasing rate of 3 °C/min. It then remained at 100 °C for 4 h. It was raised to 250 °C at a rate of 5 °C/min and then held at 250 °C for 4 h. The sample preparation was completed, and the sample was removed from the furnace and ground into a powder. The dried coal fly ash containing sodium methoxide for the subsequent transesterification reaction and the sodium methoxide solution used as a homogeneous catalyst for transesterification reaction are compared in Figure 2.

**Figure 1:**
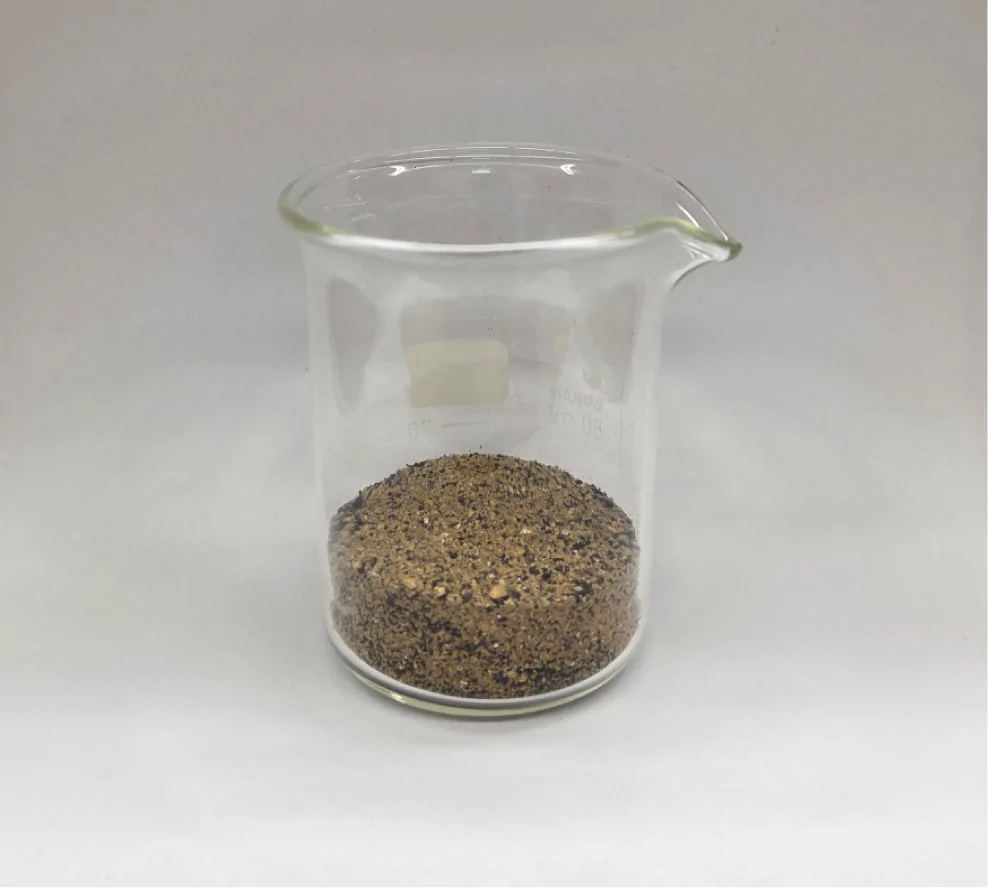
Coal fly ash used in this experiment.

**Figure 2:**
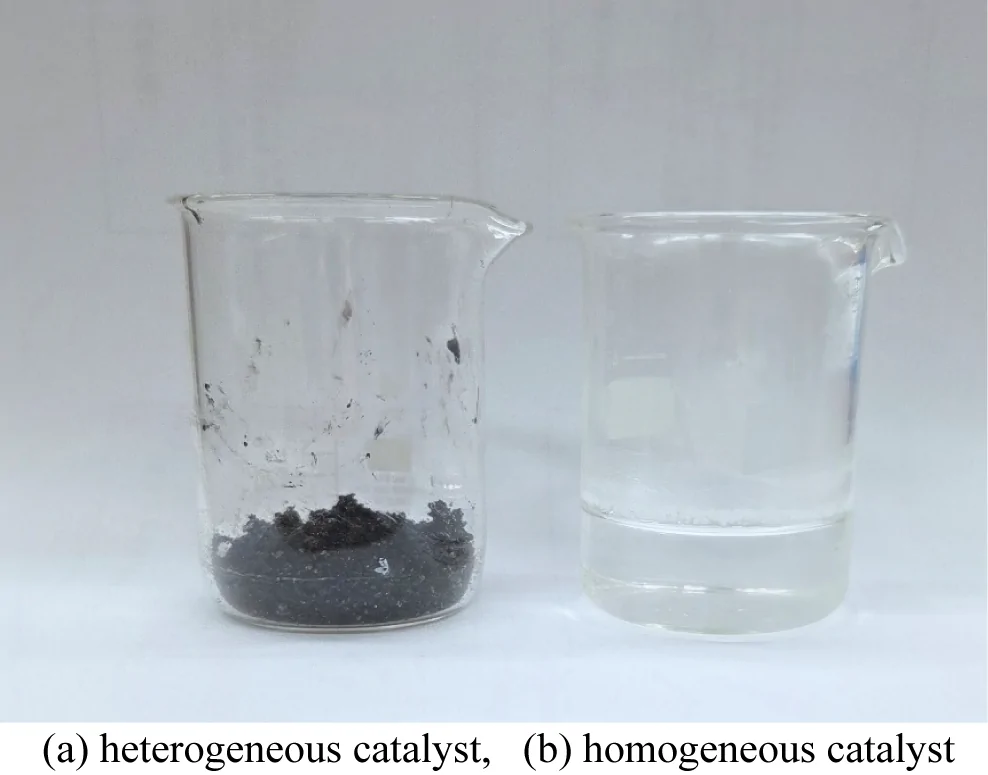
Comparison of heterogeneous and homogeneous catalysts used in the transesterification reaction for biodiesel production.

Palm oil was vended from Formosa Oilseed Processing Co., Ltd. The palm oil undergoes traditional transesterification using a homogeneous catalyst, sodium methoxide. In this study, a heterogeneous catalyst for transesterification, in which sodium methoxide was supported on fly ash, was prepared and used in the reaction. 100 mL of palm oil was added to a round-bottom flask, which was placed on a magnetic heating and stirring plate. The palm oil was preheated to 65 °C, and methanol was then added at molar ratios of 5/1 to 9/1. Fly ash/sodium methoxide mixtures at weight proportions of 1.2–7.1 wt % of reacted palm oil were prepared and then added to the flask. The heating stirrer was set to 500 rpm and 65 °C, and the reaction was run for 1 h. After the reaction was complete, the fly ash catalyst was separated from the glycerol-palm-oil biodiesel mixture. Deionized water was used to clean the biodiesel for 3 times. The biodiesel was then heated to 105 °C for 20 min to remove water, methanol, volatile compounds, and other impurities, thereby completing the biodiesel manufacturing process.

The compound composition of the biodiesel made from the transesterification reaction using homogeneous and heterogeneous catalysts was analyzed and compared using a gas chromatograph coupled to a mass spectrometer (GCMSQP2010 SE, Shimadzu Ltd., Kyoto, Japan). This implies that the GC–MS equipment is composed of Gas Chromatography and Mass Spectrometry. Liquid samples were first vaporized and introduced into a capillary column using an inert carrier gas, such as nitrogen or argon. The compounds have various chemical properties and molecular weights, and were thus separated into columns. The chemical structure of each compound was determined based on its retention time. The GC oven was initially set to 50 °C and then ramped to 270 °C. After reaching 300 °C, mass spectrometry analysis began.

### Analysis of Engine Performance and Emission Characteristics

A diesel engine (UMBDI model, Isuzu Ltd., Tokyo, Japan) was used to evaluate the performance and emission characteristics of biodiesel produced via transesterification of palm oil with heterogeneous or homogeneous catalysts. The engine is a four-stroke, in-line four-cylinder, naturally aspirated, direct-injection, freshwater-cooled marine diesel engine for a fishing boat. The engine idle speed is 500 rpm, and the compression ratio, displacement volume, and maximum output power are 17:1, 3856 cc, and 67.53 kW (88 ps) at 2800 rpm. The experimental setup for the diesel engine test is illustrated in Figure 3. An eddy current engine dynamometer (FE-150S model, Borghi & Saveri Ltd., Italy) was used to control engine torque output by connecting the transmission shaft to the diesel engine flywheel. The max. engine brake torque is 277.34 N·m, and the maximum rated power is 111.8 kW (150 hp). An engine control associated with the data acquisition system was installed to control the fuel valve opening and excitation current, thereby enabling adjustment of engine speed, output torque, and the fuel valve controller. The specifications of the eddy current engine dynamometer are shown in Table 1.^14^ The max. power output and max. torque are 150 hp and 28.3 kgf-m, respectively. When the engine is operated abnormally, such as excessive temperatures of the cooling water and lubricating oil, too low levels of the cooling water and lubricating oil, or engine overspeed, the engine alarm will be triggered automatically, reducing speed sharply or even shutting down emergently. An exhaust gas analyzer (CA-300 NSX model, Bacharach Ltd., U.S.A.) was used to measure the exhaust gas composition and concentrations from the diesel engine. The gaseous pollutants, such as CO, NOx, SO_2_, and O_2_, as well as the excess air ratio and exhaust gas temperature, were measured simultaneously. Engine efficiency can also be calculated using the analyzer’s internal calculator. Prior to the test, the dynamometer’s engine torque should be calibrated by applying counterweights to the load cell on both sides, using the formula: weight of the counterweight × its arm length =engine torque. Engine torque can be calibrated progressively by reducing the counterweight’s weight. After carefully verifying that the engine could be operated accurately, the engine test began by setting a constant engine torque of 98 N-m and adjusting the engine speed from 1200 to 1800 rpm.

**Figure 3:**
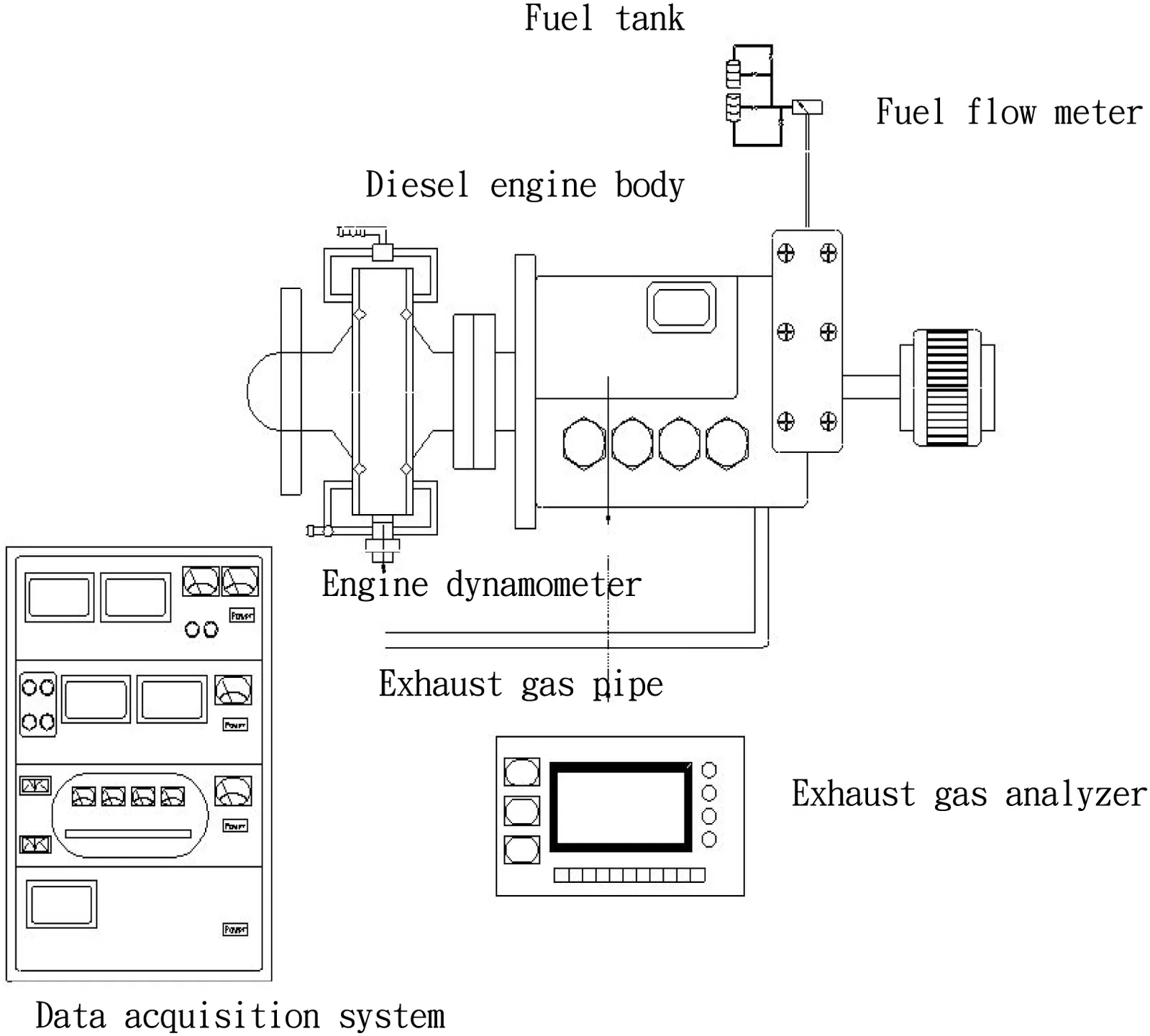
The experimental setup of a diesel engine dynamometer.

**Table 1:** Specifications of Eddy Current Dynamometer^a^

Item	Specs
Manufacturer	Borghi & Saveri
Model	FE-150S
Max. power output	150 hp
Max. torque	28.3 kgf-m
Max. speed	13000 rpm
Torque detector	precision ± 0.2%
Speed detector	60-tooth magnetic response, precision ±0.1 rpm

a Data source: Borghi & Saveri Ltd., Operating Manual of Eddy Current Dynamometer, model FE-150S, Pieve di Cento (BO), Italy, 2022.^14^

Cetane index (CI) is an alternative parameter to cetane number for indicating the difficulty extent of compression ignition of an engine fuel. The cetane index is calculated from the specific gravity (SG) and the temperature at which 50% of the liquid fuel is distilled and condensed, i.e., T_50._ The calculation formula (2) is expressed as,^15^

2 $$\mathrm{CI}=-420.34+0{.016G}^{2}+0.192G\left(\log T_{50}\right)+65.01(\log T_{50}{)}^{2}-0.0001809(T_{50}{)}^{2}$$

The unit of T_50_ in the equation is_◦_F, and G is termed as API (American Petroleum Institute) gravity, which is calculated by eq 3:

3 G=(141.5/SG)−131.5

The engine brake specific fuel consumption (BSFC) is defined as the mass flow rate of fuel consumed per unit of power output, as given by eq 4

4 $${\mathrm{Bsfc}=ṁ}_{f/P}$$

where *ṁ*_f_ is the mass flow rate of fuel consumption, in units of g/h;

P = brake power output corresponding to the fuel consumed, in units of kW.

The brake specific fuel consumption (BSFC) is used as an index of the combustion efficiency of an internal combustion engine. Lower BSFC is preferable because it indicates low fuel consumption required to produce the same brake power output.

The exhaust gas analyzer was also activated to measure the concentrations of various gaseous pollutants emitted by the engine. The mean values were obtained by repeating each experiment 3 to 5 times. The experimental uncertainties of the O_2_, CO_2_, NOx, CO, exhaust gas temperature, equivalence ratio, fuel consumption rate, BSFC (brake specific fuel consumption), brake thermal efficiency, and excess air ratio were 2.37%, 1.69%, 3.25%, 1.83%, 2.06%, 1.78%, 1.52%, 2.19%, 1.83%, and 1.46%, respectively.

There are three types of error when conducting an experiment.^16^ The first one is gross blunders in apparatus or instrument construction which may invalidate the data. The second error is fixed errors, which will cause repeated readings to be in error by roughly the same amount. The third type of error is termed random error, which may be caused by personal fluctuations, random electronic fluctuations in the apparatus or instruments, friction, etc.

## Results and Discussion

### Preparation for the Catalyst and Biodiesel

The fuel properties of biodiesel produced from palm oil using homogeneous and heterogeneous catalysts containing coal fly ash and sodium methoxide were compared. The appearance of the two biodiesels produced by the two different catalysts is shown in Figure 4. The photograph on the right-hand side shows the biodiesel produced by the heterogeneous catalyst. Soluble compounds of SiO_2_, Al_2_O_3_, and Fe_2_O_3_ are exudated from coal fly ash to the biodiesel so that the biodiesel appearance becomes darker than that of biodiesel made from a homogeneous catalyst (i.e., NaCH_3_O), as shown at the right of Figure 4. In addition, the fatty acid methyl esters of the biodiesel made from two different catalysts at various molar ratios of methanol/palm oil from 5 to 9 are compared in Table 2. The FAME yield from the heterogeneous catalyst is significantly higher, particularly when the molar ratio exceeds 7. Higher FAME percentages in biodiesel indicate a higher conversion rate from palm oil to biodiesel and a greater extent of transesterification, which may also contribute to the darker appearance of biodiesel produced with a heterogeneous catalyst.

**Figure 4:**
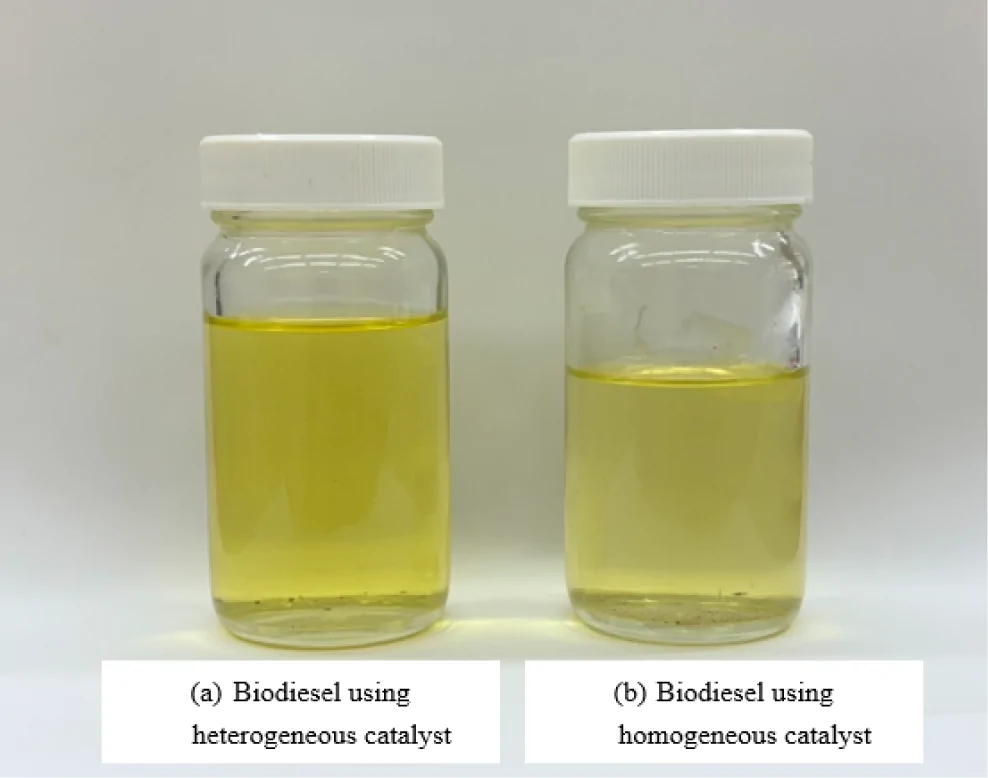
Biodiesels prepared through transesterification reaction using (a) heterogeneous catalyst and (b) homogeneous catalyst.

**Table 2:** (a) FAME (%) Formation in Biodiesel, and (b) Elemental Compositions of the Biodiesel Made Using Homogeneous and Heterogeneous Catalysts

(a)		
Molar ratio of alcohol to oil	Homogeneous catalyst of 0.5 wt %	Heterogeneous catalyst of 3.1 wt %
**5**	91.95	92.58
**6**	96.18	96.54
**7**	69.79	93.27
**8**	63.28	92.14
**9**	56.34	91.73

(b)			
	Elements (wt %)		
oil	C	H	O
SLSD	82.09	12.76	4.69
Homogeneous catalyst of 0.5 wt %	76.34	12.07	10.66
Heterogeneous catalyst of 3.1 wt %	75.97	11.96	10.405

The FAME of the biodiesel produced from a heterogeneous catalyst is higher than that from a homogeneous catalyst by 32.21% at a molar ratio of 7. The FAME from biodiesel is even higher than that of the latter biodiesel by 62.01%. At a molar ratio of 6, the biodiesel made from either heterogeneous or homogeneous catalysts appeared to have the highest FAME formation among various molar ratios from 5 to 9.

This engine-testing study used fly ash derived from coal-fired boilers as the catalyst support. Sodium methoxide was synthesized as the catalyst via impregnation, and palm oil was converted into fatty acid methyl esters (FAME) via transesterification. The resulting biodiesel was subjected to engine performance testing and exhaust emission analysis.

This experiment employed a diesel engine dynamometer and exhaust-gas measurement instruments to evaluate biodiesel engine performance and exhaust gas characteristics. The engine is a Japanese-made Isuzu Ltd. (UMBD1 model), with a displacement of 3856 cc and an inline four-cylinder, four-stroke, naturally aspirated diesel engine. Engine output torque and horsepower are controlled using an Italian-made Borghi & Saveri eddy-current dynamometer with a maximum horsepower of 111.8 kW (150 hp) and a maximum torque of 277.34 N·m.

The tested fuels include biodiesel produced with a methanol-to-oil molar ratio of 6/1 and a heterogeneous catalyst concentration of 2.8 wt %, biodiesel produced with a homogeneous catalyst concentration of 0.5 wt %, and commercial super low sulfur diesel (SLSD). The experiments were conducted under a fixed torque condition (98 N·m) at varying engine speeds from 800 to 2000 rpm. The experimental results are presented and discussed as follows.

### Effects of Engine Speed and Fuel Type on Fuel Consumption Rate

The fuel consumption rate increased proportionally with rising engine speed because, to maintain constant engine torque, horsepower and mechanical efficiency increased accordingly, resulting in greater fuel injection quantity and thus a higher fuel consumption rate. Furthermore, when the fuel’s calorific value is higher, the fuel consumption rate is lower to maintain the same engine power output. As shown in Figure 5, the fuel consumption rate of super low sulfur diesel (SLSD) is the lowest. According to Table 1, the calorific value of SLSD is 46.10 MJ/kg, which is the highest among the three fuel types. Therefore, its fuel consumption rate is the lowest among the three fuel samples. In contrast, biodiesel produced with a 0.5 wt % homogeneous catalyst exhibited the highest fuel consumption rate because it has the lowest heating value among the three fuel samples in Table 3. The results agree well with those of Wu et al.^17^ At an engine speed of 1600 rpm, the biodiesel produced with a homogeneous catalyst consumed 18.91% more fuel than SLSD.

**Figure 5:**
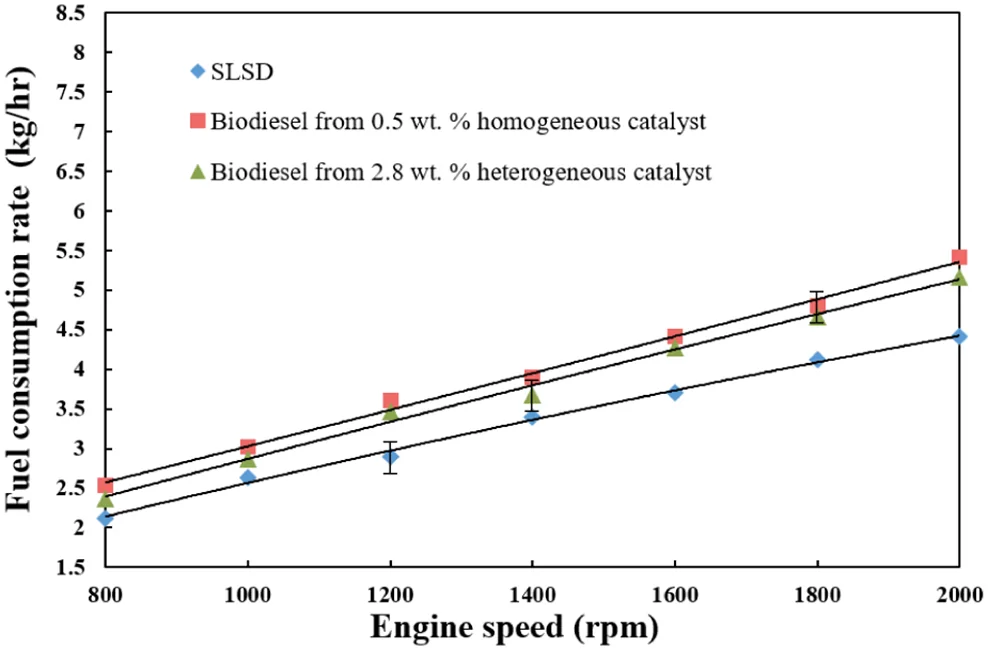
Fuel consumption rate under constant engine torque and varying engine speeds.

**Table 3:** Fuel Properties of Engine Test Fuels

	Fuel type		
Fuel properties	SLSD (super low sulfur diesel)	Biodiesel made from 0.5 wt.% homogeneous catalyst	Biodiesel made from 2.8 wt.% heterogeneous catalyst
FAME (wt %)	--------	96.18	96.54
Heating value (MJ/kg)	46.18	39.84	40.12
Kinematic viscosity (mm^2^/s, at 40 °C)	3.61	4.11	4.32
Specific gravity	0.836	0.878	0.872
Acid value (mg KOH/g)	---------	0.28	0.31
Water content (wt %)	0.006	0.039	0.038
Cetane index	59.01	47.68	49.35
Density (kg/m^3^)	835	878	872

### Effects of Engine Speeds and Fuel Type on Brake Specific Fuel Consumption (BSFC)

From Figure 6, it is observed that BSFC decreased slightly as engine speed increased. A decrease in BSFC signifies that the increase in fuel consumption rate was less than that of brake power^18^ as engine speed rose. In Figure 2, SLSD has the lowest BSFC because it has the highest calorific value among the three fuels. To achieve the same engine brake power, a lower fuel consumption rate is therefore required for the SLSD fuel. As shown in Table 3, biodiesel produced with a heterogeneous catalyst has a higher heating value than biodiesel produced with a homogeneous catalyst.^19^ This implies that, at the same brake power, the fuel consumption rate of biodiesel produced by the heterogeneous catalyst is lower than that of biodiesel produced by the homogeneous catalyst. Consequently, the BSFC of the biodiesel made using a heterogeneous catalyst appeared to be 1.95% lower than that using a homogeneous catalyst. This is consistent with the engine testing and exhaust emission characteristics reported by Bhan et al.^20^

**Figure 6:**
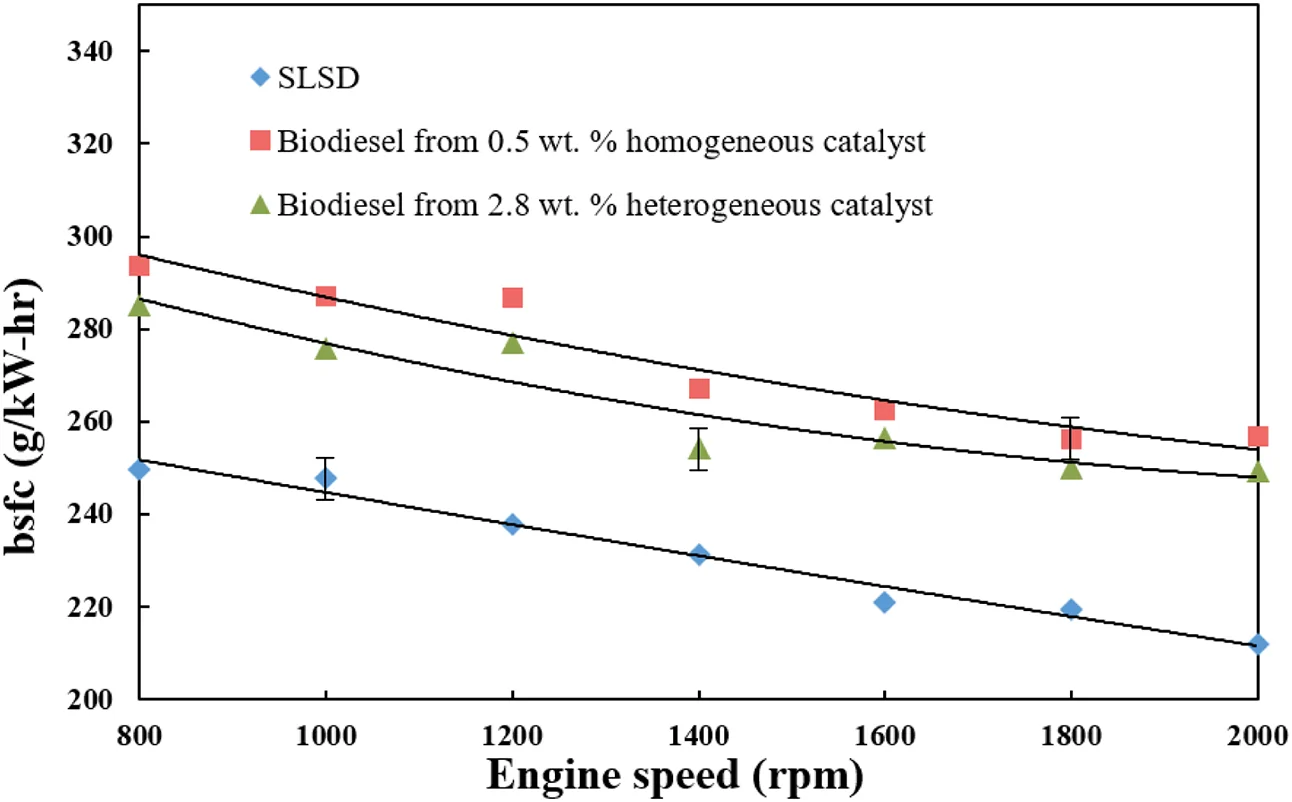
BSFC of the sample fuels under constant engine torque and varying engine speeds.

### Effects of Engine Speeds and Fuel Type on Brake Thermal Efficiency

Brake thermal efficiency is defined as the ratio of work produced per cycle during engine operation to the heat supply rate of the fuel, as shown in eq 5:

5 $$\eta_{f}=\frac{P_{b}}{\overset{˙}{m_{f}}Q_{HV}}=\frac{3600\left(kW\right)}{bsfc\left(\frac{g}{kW\cdot h}\right)Q_{HV}\left(\frac{MJ}{kg}\right)}$$

In which η_f_ represents the brake thermal efficiency, and P_b_ represents the brake horsepower. *ṁ_f_* indicates the fuel consumption rate, and Q_HV_ is the heating value of the fuel. Figure 7 compares brake thermal efficiency across three fuel types at a fixed torque and varying engine speeds. As shown in Figure 7, brake thermal efficiency increased slightly with engine speed, consistent with the observation that higher engine speed enhances thermal efficiency,^21^ thereby reducing fuel consumption. From eq 1, BSFC decreased as the engine speed increased, leading to an increase in the brake thermal efficiency.

**Figure 7:**
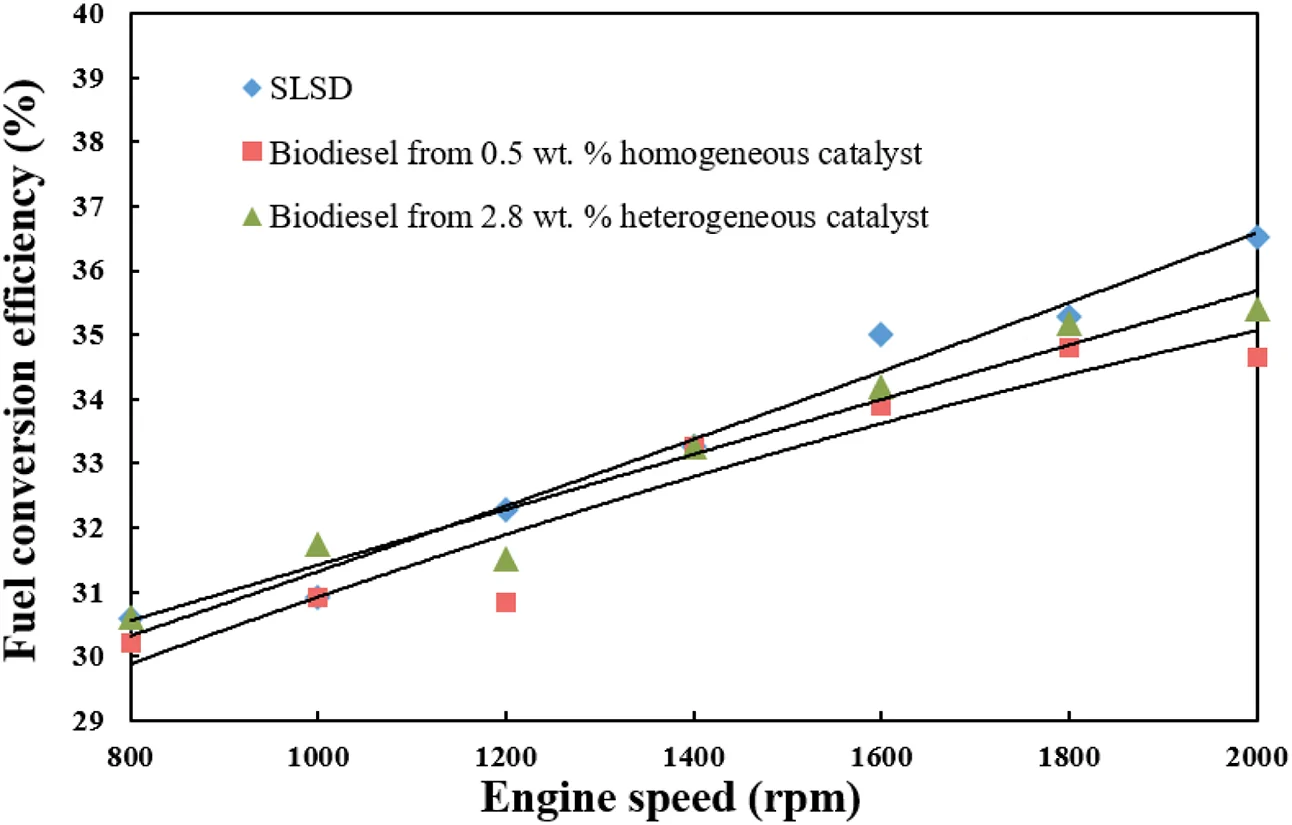
Brake thermal efficiency under constant engine torque and varying engine speeds.

Hence, the brake thermal efficiency increased with engine speed. As shown in Figure 7, at engine speeds below 1200 rpm, the brake thermal efficiency of SLSD is slightly lower than that of biodiesel produced with a 2.8 wt.% heterogeneous catalyst. However, when the engine speed exceeded 1200 rpm, the brake thermal efficiency of the SLSD exceeded that of biodiesel produced with a 2.8 wt.% heterogeneous catalyst. Lin et al.^22^ found that, at low engine speeds, biodiesel’s higher oxygen content increases its combustion efficiency relative to SLSD. As a result, the brake thermal efficiency of the biodiesel from a heterogeneous catalyst is higher than that of SLSD. Nevertheless, as engine speed increases, turbulence in the burning gases within the combustion chamber intensifies, thereby enhancing combustion efficiency. The improvement in combustion efficiency attributable to biodiesel’s oxygen content is further reduced as engine speed increases; the brake thermal efficiency of biodiesel is slightly lower than that of SLSD. The heating value of the biodiesel from a heterogeneous catalyst is higher than that of the biodiesel from a homogeneous catalyst in Table 1. Therefore, the brake thermal efficiency of the former biodiesel is higher compared to the biodiesel from a homogeneous catalyst. This was consistent with the engine performance and exhaust gas emissions results for SLSD and biodiesel reported by Lai et al.^23^

### Effect of Engine Speed and Fuel Type on O_2_ Emission Concentration

As shown in Figure 8, the O_2_ emission concentration decreased with increasing engine speed because the engine’s fuel consumption rate increased. More O_2_ was required for combustion, which resulted in a lower O_2_ emission concentration. In Table 4, biodiesel had a higher oxygen content, which can enhance its combustion characteristics.^24^ Therefore, in Figure 8, the O_2_ emission concentrations of those two biodiesels were higher than that of SLSD. Furthermore, as shown in Table 4, the oxygen content of the biodiesel from a homogeneous catalyst was higher than that of the biodiesel from a heterogeneous catalyst, resulting in a higher O_2_ emission concentration for the former biodiesel.^25^ The O_2_ emission concentration from burning biodiesel with a heterogeneous catalyst is 0.73% lower at an engine speed of 1600 rpm.

**Figure 8:**
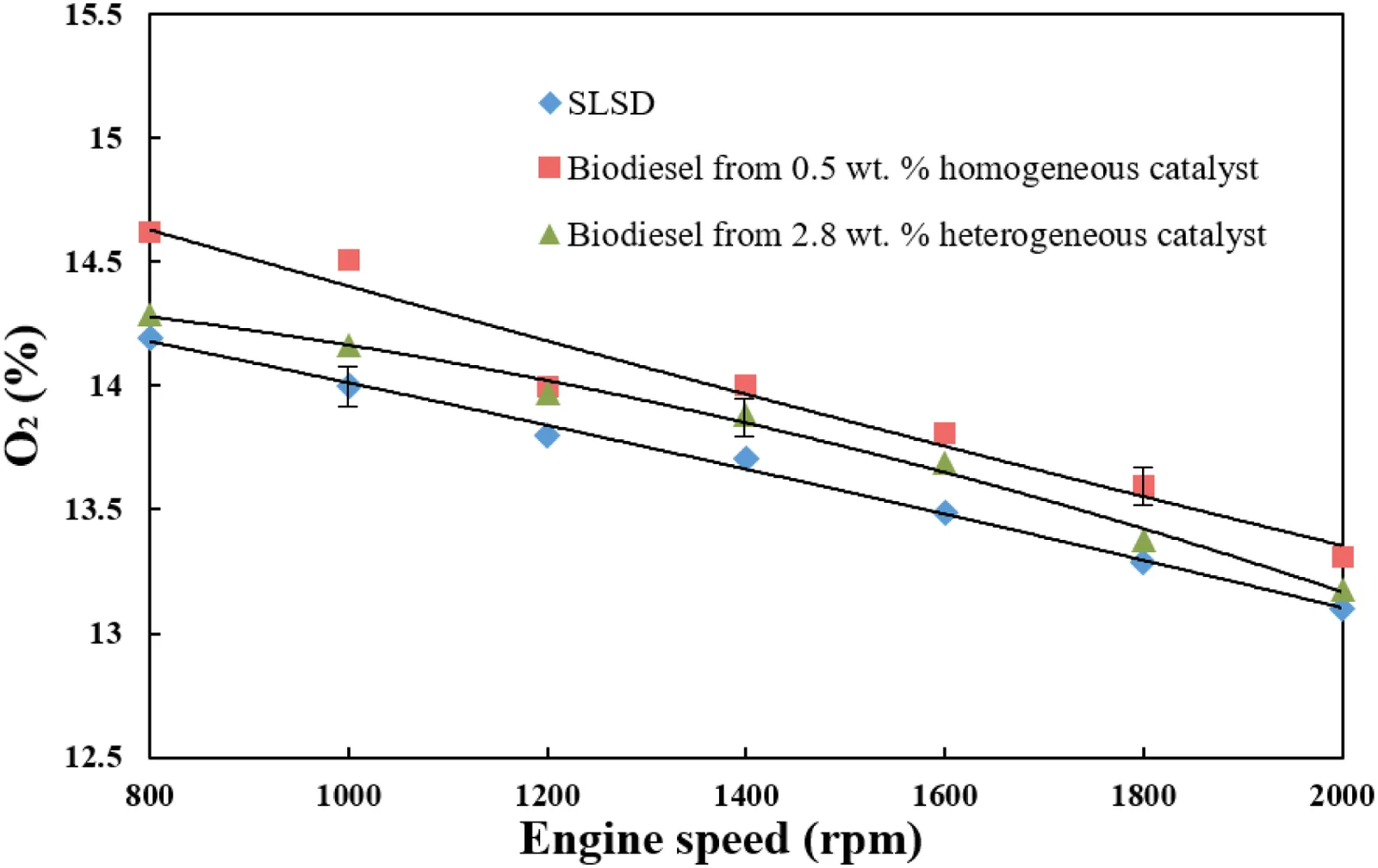
O_2_ emission concentration under constant engine torque and varying engine speeds.

**Table 4:** Elemental Compositions (Wt %) for Engine Test Fuels

	Element		
Fuel type	C	H	O
SLSD (super low sulfur diesel)	86.78	12.76	0.0
Biodiesel made from 0.5 wt % homogeneous catalyst	76.34	12.07	10.66
Biodiesel made from 2.8 wt % heterogeneous catalyst	75.97	11.96	10.405

### Effects of Engine Speed and Fuel Type on Excess Air Ratio

The excess air ratio (%) is expressed by eq 6:

6 $$\%\;excess\;air=\frac{100\left(m_{a}-m_{as}\right)}{m_{as}}=\frac{100\left(n_{o_{2}}-n_{o_{2\left(s\right)}}\right)}{n_{o_{2\left(s\right)}}}$$

Where $m_{a},m_{as,}n_{o_{2},}n_{o_{2\left(s\right)}}$ represent the mass of actual air, the mass of stoichiometric air, the molar number of actual oxygens, and the molar number of stoichiometric oxygens, respectively.

As shown in Figure 9, the excess air ratio is inversely proportional to engine speed.^26^ This is because the diesel engine operates on compression ignition, which requires compressing and heating the air-fuel mixture to initiate ignition. At low engine speeds, the required fuel consumption rate is low, resulting in a lower air requirement for combustion. It follows that the excess air ratio increased. Conversely, as engine speed increased, the fuel consumption rate also rose. The amount of air required for combustion increased, resulting in a lower excess air ratio. Therefore, the excess air ratio decreases with increasing engine speed in Figure 9. The excess air ratio is calculated from the O_2_ concentration measured by the exhaust gas analyzer; a higher O_2_ concentration corresponds to a greater excess air ratio. As shown in Figure 8 and Table 4, the O_2_ concentrations in biodiesels produced with homogeneous and heterogeneous catalysts were higher than in SLSD, indicating that SLSD required less air for combustion. Consequently, Figure 9 shows that the excess air ratio of SLSD is lower than that of the biodiesels. Because the O_2_ emission concentration and oxygen content of the biodiesel from the homogeneous catalyst exceeded those of the biodiesel from the heterogeneous catalyst, the excess air ratio of the biodiesel from the homogeneous catalyst is higher than that of the biodiesel from the heterogeneous catalyst.^27^ In fact, the excess air of the former biodiesel exceeded that of the latter biodiesel by 1.78% at 1800 rpm engine speed.

**Figure 9:**
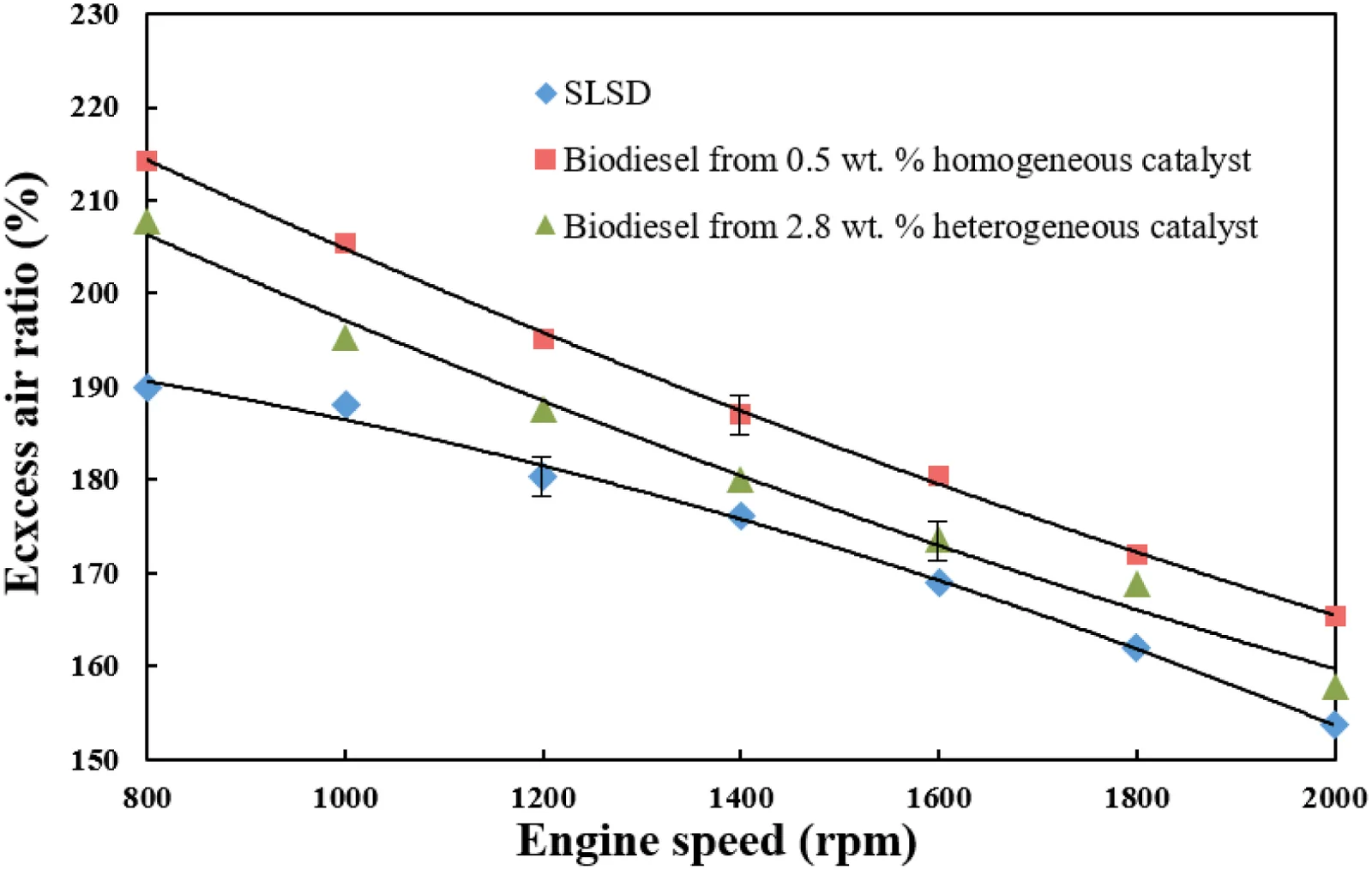
Excess air ratio under constant engine torque and varying engine speed.

### Effects of Engine Speed and Fuel Type on Fuel/Air Equivalence Ratio

The formula for the fuel/air equivalence ratio is presented in eq 7:

7 ψ=1/(excess air %+1)

In the equation, ψ denotes the fuel/air equivalence ratio, and excess air represents the excess air ratio (%). As shown in Figure 10, the fuel/air equivalence ratio tended to increase with engine speed^28^ because higher engine speeds increase fuel consumption, thereby raising the equivalence ratio. Owing to the higher oxygen content of the biodiesel, the oxygen consumed during combustion inside the engine cylinder is relatively lower than that of SLSD.^29^ Hence, the required amount of air is reduced. As a result, the excess air ratio increased due to reduced air mass consumption, resulting in a lower fuel/air equivalence ratio than that of SLSD. In Figure 10, the fuel/air equivalence ratio of the biodiesel from a homogeneous catalyst is lower than that of the biodiesel from a heterogeneous catalyst by 0.22% at an engine speed of 1600 rpm because the oxygen content of the biodiesel from a homogeneous catalyst is higher than that of the biodiesel from a heterogeneous catalyst, and the equivalence ratio shows an opposite trend to the excess air ratio in comparison with Figures 9 and 10.

**Figure 10:**
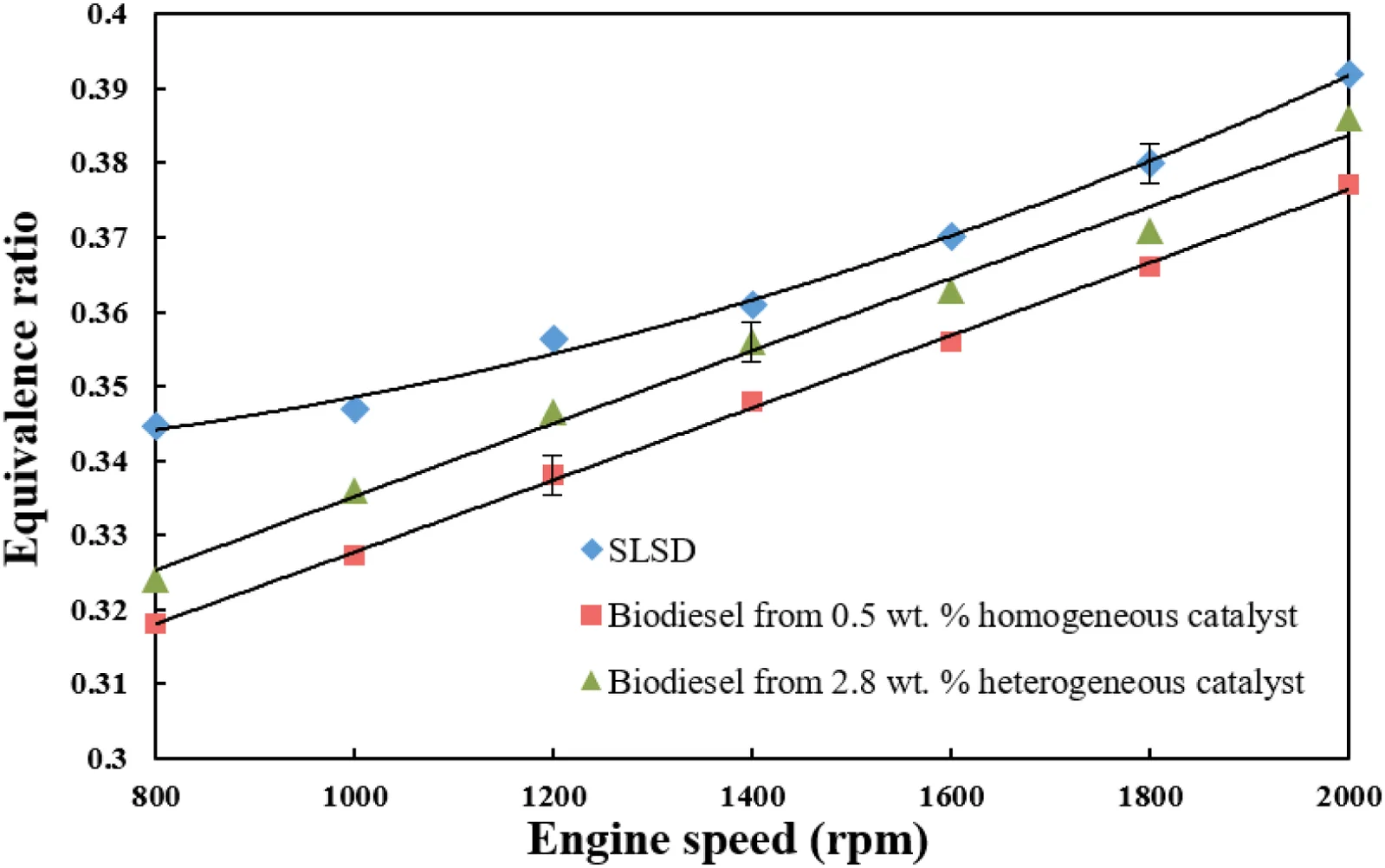
Fuel/air equivalence ratio under constant engine torque and varying engine speeds.

### Effects of Engine Speed and Fuel Type on Exhaust Gas Temperature

Exhaust gas temperature rose with increasing engine speed due to the greater corresponding heat released from the fuels. According to Table 3 and Figure 11, superlow-sulfur diesel (SLSD) has the highest heating value among the three fuel samples. Therefore, its exhaust gas temperature is correspondingly higher. The exhaust gas temperatures of those two biodiesels are lower than that of SLSD. Guo et al.^30^ attributed this to the higher oxygen content of biodiesel, which promotes combustion and yields superior cylinder-level performance compared with SLSD. Moreover, biodiesel has a lower heating value than SLSD, leading to a lower exhaust gas temperature. Moreover, as shown in Figure 11, the exhaust gas temperature of biodiesel from a heterogeneous catalyst is slightly higher than that of biodiesel made from a homogeneous catalyst by 2.46% because the heating value of the biodiesel from a heterogeneous catalyst is higher than that of biodiesel from a homogeneous catalyst by 0.7%, as presented in Table 3.

**Figure 11:**
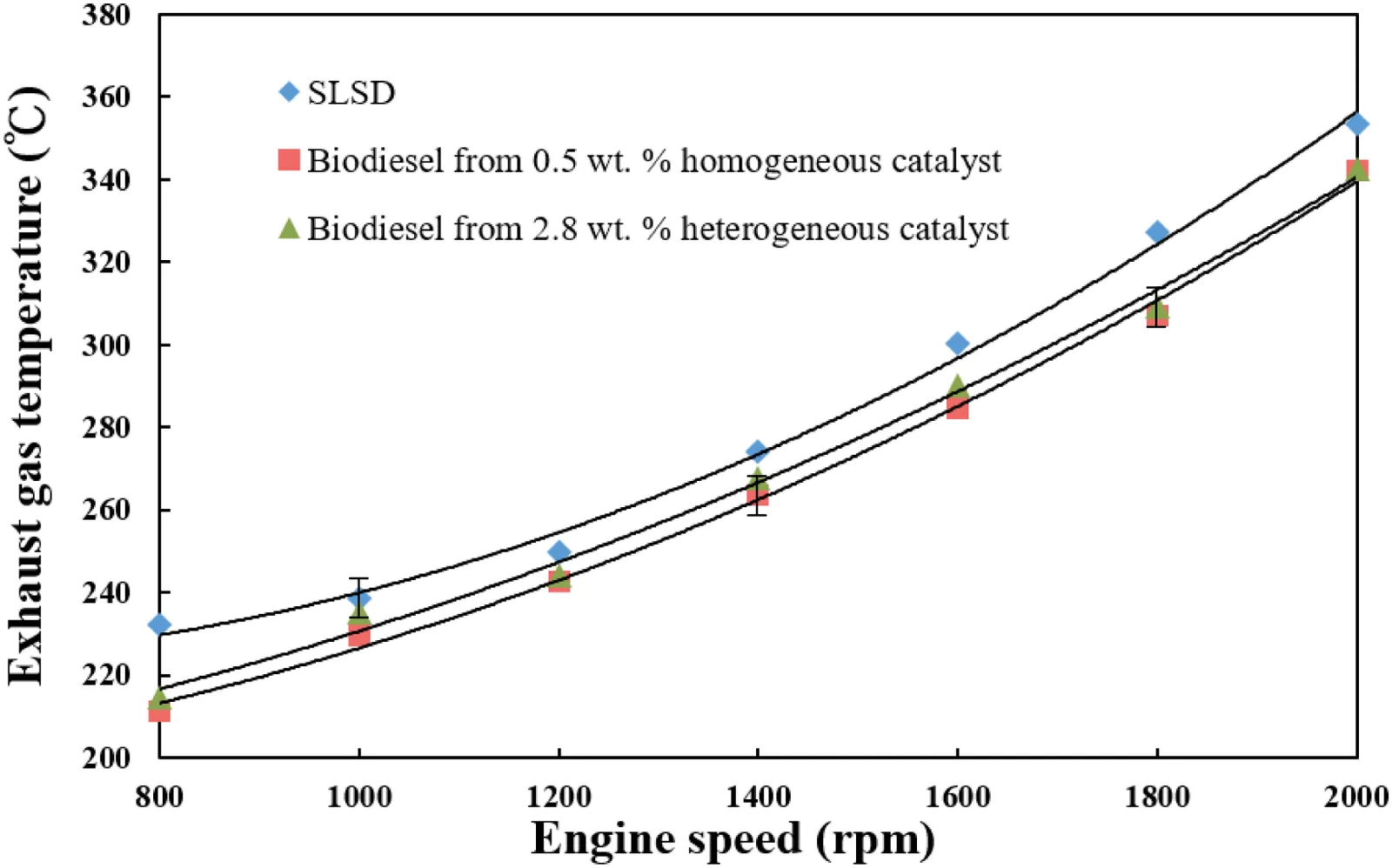
Exhaust gas temperatures for three fuel samples under constant engine torque and varying engine speeds.

### Effect of Engine Speed and Fuel Type on CO Emission Concentration

As shown in Figure 12, the CO emission concentration decreased with increasing engine speed. This is because the CO emission concentration is related to the gas temperature inside the engine cylinder. At low engine speeds, the gas temperature inside the engine cylinder is lower, resulting in a longer CO oxidation time, which prevents complete oxidation of CO to CO_2_ and increases CO emission concentration.^31^ Equations 8 and 9 represent the CO oxidation reaction and the calculation formula for the CO oxidation rate:

8 $$CO+OH↔CO_{2}+H$$

9 $$K_{CO}^{+}\left(\frac{cm^{3}}{gmol}\right)=6.76\times 10^{10}\exp\left[\frac{T}{1104}\right]$$

**Figure 12:**
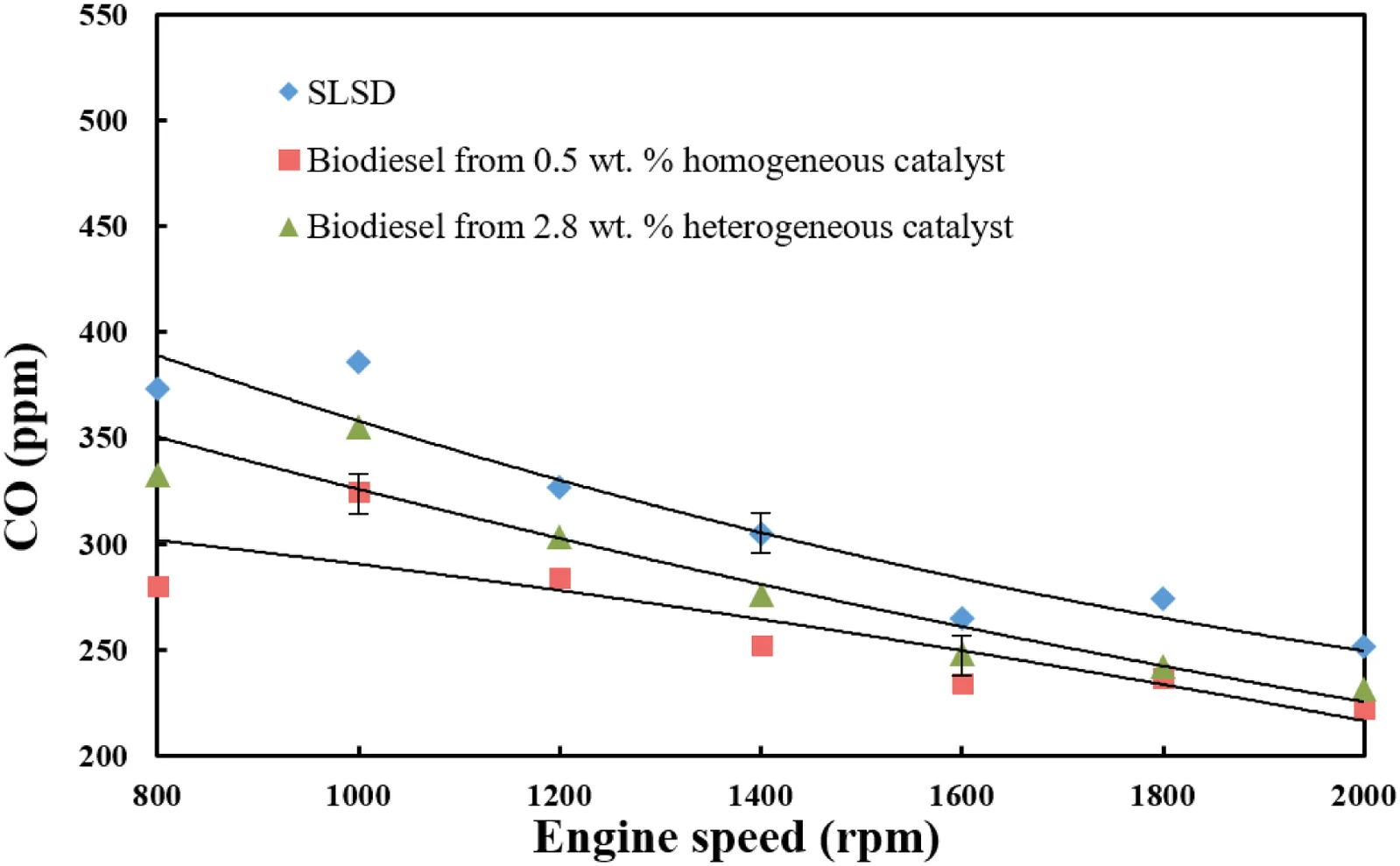
CO emission concentration under constant engine torque and varying engine speed.

in which K^+^_CO_ represents the CO oxidation rate, and T is the burning gas temperature, measured in K. eq 5 shows that as the temperature T increases, K^+^_CO_ increases as well. A higher CO oxidation rate corresponds to a lower CO emission concentration. Therefore, the engine produces lower CO emissions at higher engine speeds. As shown in Figure 12, both biodiesels made from heterogeneous and homogeneous catalysts yielded lower CO emissions than SLSD.^32^ Table 4 shows that biodiesel is an oxygen-rich fuel, which enhances combustion and promotes the oxidation of CO within the engine cylinder, resulting in reduced CO emission concentrations for both biodiesels. In addition, the CO emission concentration from the biodiesel made from a homogeneous catalyst is lower than that of the biodiesel from a heterogeneous catalyst by 13.67%. As observed from Figure 8 and Table 4, both the O_2_ emission and elemental oxygen content in the biodiesel from a homogeneous catalyst are higher than those of the biodiesel from a heterogeneous catalyst. Hence, the CO emission concentration is lower in the former biodiesel.

### Effects of Engine Speed and Fuel Type on CO_2_ Emission Concentration

Figure 13 shows that CO_2_ emission concentration increases with engine speed, as it is proportional to the fuel/air equivalence ratio shown in Figure 10. When the equivalence ratio increases, the CO_2_ emission concentration also rises.

**Figure 13:**
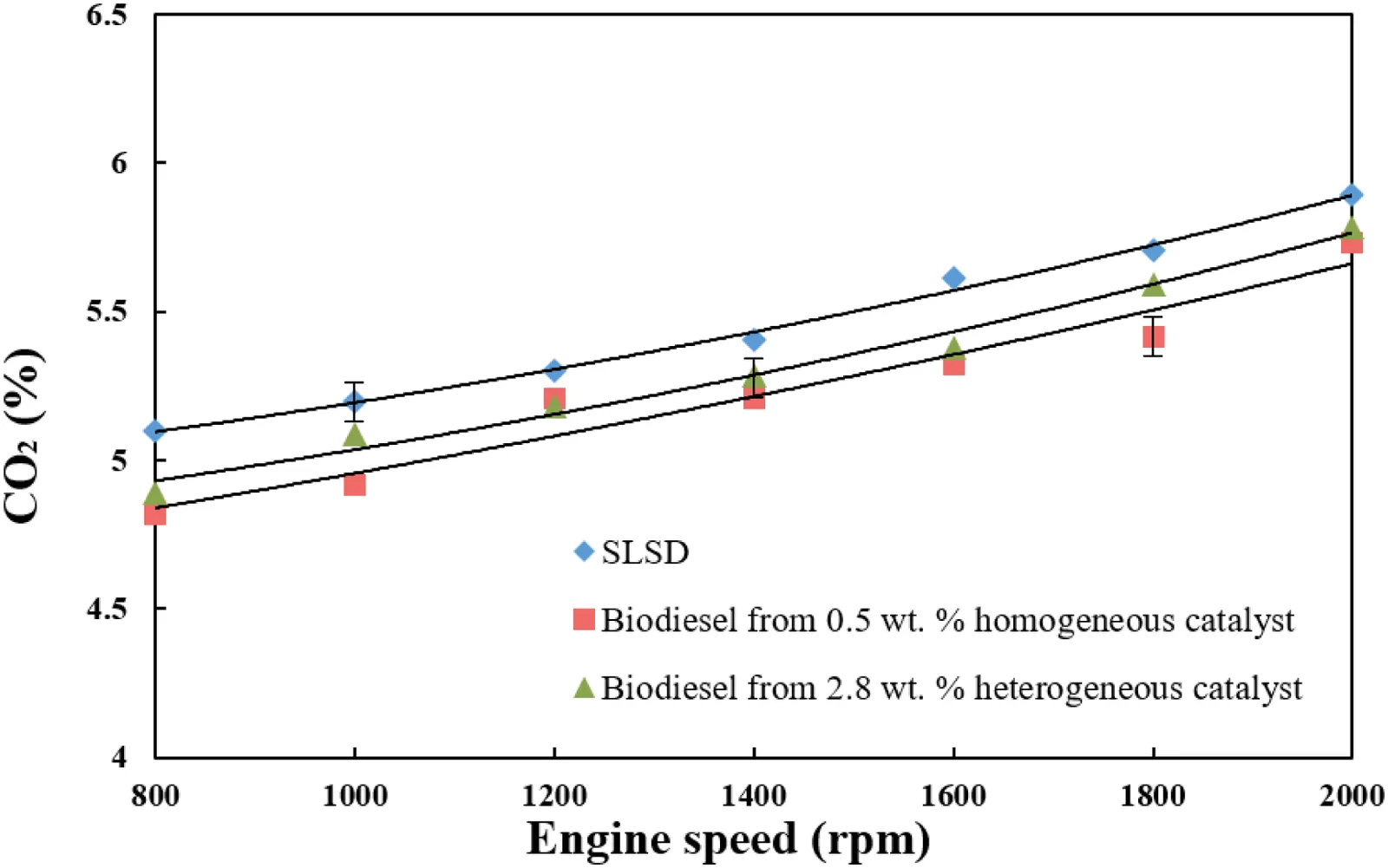
CO_2_ emission concentration under constant engine torque and varying engine speeds.

In Figure 13, the CO_2_ emission concentrations of both biodiesels produced with heterogeneous and homogeneous catalysts are lower than those of SLSD, as CO_2_ emission concentrations increase with the fuel/air equivalence ratio and elemental carbon content. As shown in Figure 10, the fuel/air equivalence ratio of the biodiesel is lower than that of SLSD, which has a higher elemental carbon content. Therefore, SLSD exhibited higher CO_2_ emission concentrations. Moreover, the fuel/air equivalence ratio of the biodiesel from a heterogeneous catalyst is higher than that of the biodiesel from a homogeneous catalyst. Consequently, the CO_2_ emission concentration of the former biodiesel is higher by 1.88% at an engine speed of 1600 rpm, which aligns with the engine test speed and exhaust gas characteristic results for premium diesel and biodiesel reported by Allasi et al.^33^

### The Effect of Engine Speed and Fuel Type on NO_X_ Emission Concentration

It can be observed from Figure 14 shows that NO_X_ emission concentration decreased as engine speed increased. This is because at high engine speeds, the volumetric efficiency of the engine cylinders improves, and the gas velocity in the cylinders increases as well,^34^ leading to accelerated mixing of fuel and air and, in turn, a shortened ignition delay. As the combustion reaction accelerated, the residence time of high-temperature gases in the cylinder decreased. When the gases contact cooler gases or air within the cylinder, the gas temperature drops, thereby reducing the NO_X_ emission concentration.

**Figure 14:**
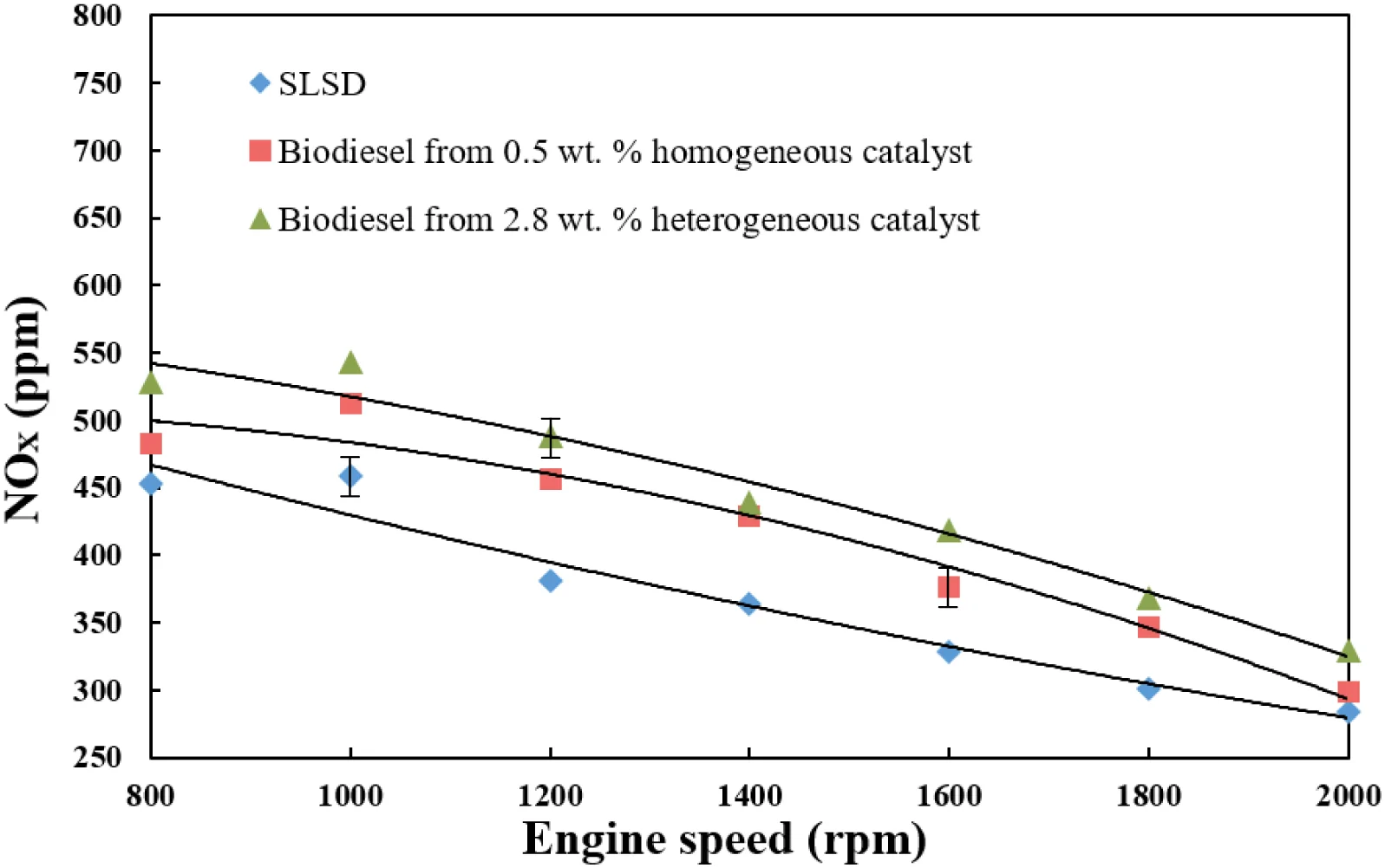
NOx emission concentration under constant engine torque and varying engine speed.

In Figure 14, the NO_X_ emission concentrations of both biodiesels made with homogeneous and heterogeneous catalysts are higher than those of SLSD.^35^ This is because the higher oxygen content of the biodiesel facilitated the formation of nitrogen oxides from atmospheric nitrogen at elevated temperatures. A lower fuel-to-air equivalence ratio indicates fuel-lean combustion in a diesel engine. Under fuel-lean combustion, NO_X_ emission concentrations tended to decrease until combustion quality deteriorated. Therefore, there is a combustion period prior to combustion-induced quality degradation, during which NO_X_ emission concentrations can be reduced. Figure 10 shows that the fuel/air equivalence ratio of the biodiesel from a heterogeneous catalyst is higher than that of the biodiesel from a homogeneous catalyst because the heating value of the former biodiesel is higher. This causes a higher combustion gas temperature inside the cylinder for biodiesel from a heterogeneous catalyst than for biodiesel from a homogeneous catalyst, resulting in 12.56% higher NO_X_ emissions when burning the former biodiesel. These results align with the engine test and exhaust characteristic findings of SLSD and biodiesel reported by Murad.^36^

## Conclusions

Subsequent analyses of engine performance and exhaust emissions were conducted. The experimental results are summarized as follows.

1. Among the three fuels, super low sulfur diesel (SLSD) exhibited the highest heating value (approximately 46.18 MJ/kg), resulting in a lower fuel consumption rate than the other two biodiesels.
2. Since the heating value of the biodiesel made from a heterogeneous catalyst is higher than that of the biodiesel made from a homogeneous catalyst, the bsfc value of the former biodiesel is correspondingly lower.
3. Because the heating value of the biodiesel from a heterogeneous catalyst is higher than that of the biodiesel from a homogeneous catalyst, the brake thermal efficiency of the former biodiesel is also greater than that of the latter biodiesel.
4. The biodiesel from a homogeneous catalyst having the highest oxygen content appeared to have the highest O_2_ emission, while SLSD had the lowest O_2_ emission.
5. The excess air ratio of the biodiesel from a heterogeneous catalyst is lower than that of the biodiesel from a homogeneous catalyst. Consequently, the fuel/air equivalence ratio of the former biodiesel is higher.
6. The exhaust gas temperature of the biodiesel from a heterogeneous catalyst is higher than that of the biodiesel from a homogeneous catalyst because of a higher heating value of the former biodiesel
7. The biodiesel made with a homogeneous catalyst had lower CO emissions due to its higher oxygen content than biodiesel made with a heterogeneous catalyst.
8. The fuel/air equivalence ratio of the biodiesel from a heterogeneous catalyst is higher than that of the biodiesel from a homogeneous catalyst; the CO_2_ emissions from the former biodiesel were higher.
9. The heating value of the biodiesel from a homogeneous catalyst is higher than that of biodiesel from a heterogeneous catalyst, resulting in a higher burning gas temperature of the former biodiesel, leading to its higher NO_X_ emission.

## Data Availability

All data generated or analyzed during this study are included in this published article.
